# Plant-parasitic nematodes associated with sugarcane in Kilimanjaro, Tanzania

**DOI:** 10.21307/jofnem-2020-059

**Published:** 2020-07-06

**Authors:** Phougeishangbam Rolish Singh, Beatrice E. Kashando, Marjolein Couvreur, Gerrit Karssen, Wim Bert

**Affiliations:** 1 Nematology Research Unit, Department of Biology, Ghent University, K.L. Ledeganckstraat 35, 9000 Ghent, Belgium; 2 National Plant Protection Organization, Wageningen Nematode Collection, P.O. Box 9102, 6700 HC Wageningen, The Netherlands; 3 Tanzania Agricultural Research Institute (TARI), P.O. Box 30031, Tumbi Road, Kibaha Coast region, Tanzania

**Keywords:** 28S, ITS, *COI*, TPC, Kilimanjaro, Phylogeny, Plant-parasitic nematodes, *Rotylenchulus parvus*, Sugarcane, Synonymy, *Tylenchorhynchus agri*, *T. crassicaudatus*, Tanzania, Taxonomy

## Abstract

Morphological and molecular analyses of plant**-**parasitic nematodes (PPN) from 12 sugarcane plantation sites of Tanganyika Planting Company (TPC) Limited in Kilimanjaro region of Tanzania revealed the presence of six PPN genera, i.e. *Helicotylenchus*, *Hemicycliophora*, *Pratylenchus*, *Rotylenchulus*, *Scutellonema*, and *Tylenchorhynchus*. The genera with the highest densities and present in virtually all samples were *Pratylenchus* and *Rotylenchulus*, and the most important species appeared to be *R. parvus*, *P. zeae*, *T. crassicaudatus*, and *T. ventrosignatus*. A total sequences of 11 partial ITS, 15 D2-D3 of 28S, and 6 partial 18S of rRNA gene, and 7 partial *COI* gene of mtDNA of these species were obtained in this study. Morphology and molecular data comparisons between the Tanzanian *R. parvus* and the South African *R. parvus* indicated that *R. parvus* is a cryptic species complex. Based on the results of morphological and molecular analyses of *T. crassicaudatus* and *T. agri* from China, Haiti, Indonesia, Iran, Niger and the USA, *T. agri* syn. n. is proposed as a junior synonym of *T. crassicaudatus*.

Sugarcane is an important cash crop in Tanzania which is widely used for the production of sugar for home consumption and commercial industries. Other by**-**products such as bagasse and molasses are also used as a renewable source of energy and for exporting (Arndt et al., 2010). The most important sugarcane cultivating regions in Tanzania are Morogoro (Kilombero Sugar Company and Mtibwa Sugar Estates), Kagera (Kagera Sugar Limited), and Kilimanjaro (Tanganyika Planting Company (TPC) Limited). However, sugar consumption of Tanzania is usually higher than its production, often resulting in need for import of sugar, and the country’s production of sugarcane per hectare is also reported to be lower compared to other countries such as Kenya and South Africa (Tarimo and Takamura, 1998; Songela and Maclean, 2008; Sulle et al., 2014; Sambuo, 2015).

Low sugarcane production in Tanzania has often been linked to several factors including pest and diseases like smut disease caused by fungi and white scale pest (Greathead, 1970; Msechu and Keswani, 1978; Katundu and Ramadhani, 1988). Other studies reported the effect of the white grubs, *Cochliotis melolonthoides* (Coleoptera: Scarabaeidae), which feed underground on sugarcane roots (Jepson, 1956; Cock and Allard, 2013), and accounted for an annual economic loss of about 25 to 50% at the TPC Limited, Kilimanjaro (Paray et al., 2012). However, no investigation on the diversity and the effect of nematodes on sugarcane of Tanzania has been done despite the fact that plant**-**parasitic nematodes (PPN) can damage roots and reduce the length of cane stalks leading to sugarcane yield loss (Berry et al., 2008). Several studies have uncovered PPN diversity in sugarcane fields in countries such as Mauritius (Williams, 1960a, b), India (Sundararaj and Mehta, 1993), Kenya (Chirchir et al., 2008, Steven et al., 2014), South Africa (Berry et al., 2017), and Brazil (Noronha et al., 2017), revealing the most common PPN genera associated with sugarcane as *Meloidogyne*, *Pratylenchus*, *Tylenchorhynchus*, *Rotylenchulus*, and *Helicotylenchus*.

This study was aimed at studying the diversity of PPN associated with sugarcane, with focus on the sugarcane plantation sites of TPC Limited in Kilimanjaro region. The combination of morphological and molecular analyses revealed the presence of several genera of PPN in the soil and root samples collected from 12 different sugarcane fields. Molecular and morphological characterizations are provided for the most important PPN species detected.

## Materials and methods

### Sampling and nematode extraction

Soil and root sampling was done at the end of July 2017 from 12 sugarcane fields at the TPC Limited in Kilimanjaro region, Tanzania, which is located about 50 km to the South of Mount Kilimanjaro, and 20 km from Moshi Municipality. Three soil samples from the North, five from the East, and four samples from the Southern parts of the TPC estate were collected (Table 1). The Southern fields of the TPC estate had been known to be infected by white grubs below-ground and have been treated by pesticides before soil sampling. From each site, random soil samples from a depth of about 20 to 30 cm were taken using a shovel, mixed to obtain a total volume of about 500cc of soil and, subsequently, stored at 4°C until nematode extraction. Nematodes were extracted from 100cc of soil from each collection site using a modified Baermann method (Whitehead and Hemming, 1965). Roots were also collected along with the soil and only merely checked for the presence of nematodes, also by using the modified Baermann method.

**Table 1.** Number of plant-parasitic nematodes of six different genera counted in nematode extract from 100cc of soil from 12 sugarcane plantation sites at Tanganyika Planting Company Limited in Kilimanjaro region of Tanzania in July, 2017.

Collection sites	GPS coordinates	Rotylenchulus	Pratylenchus	Tylenchorhynchus	Scutellonema	Hemicycliophora	Helicotylenchus
N50-North	3° 25´ 1.20˝ S, 37° 18´ 49.68˝ E	33	18	0	5	0	0
N54-North	3° 25´ 20.32˝ S, 37° 18´ 49.68˝ E	117	6	3	0	0	1
N84-North	3° 23´ 58.34˝ S, 37° 20´ 1.43˝ E	14	13	0	0	0	0
D8-East	3° 28´ 1.38˝ S, 37° 20´ 15.04˝ E	7	11	0	0	0	0
D30-East	3° 30´ 11.12˝ S, 37° 20´ 56.86˝ E	21	11	0	0	0	0
C6-East	3° 28´ 2.25˝ S, 37° 19´ 33.68˝ E	12	2	0	0	5	1
D20-East	3° 29´ 1.62˝ S, 37° 20´ 42.02˝ E	12	20	1	1	7	1
E11-East	3° 28´ 58.72˝ S, 37° 21´ 0.07˝ E	22	84	1	0	0	0
F13-South	3° 33´ 44.52˝ S, 37° 18´ 51.03˝ E	1037	11	5	0	0	0
F10-South	3° 31´ 9.67˝ S, 37° 20´ 15.71´ E	170	0	16	0	1	0
11E-South	3° 28´ 28.45˝ S, 37° 20´ 44.26˝ E	7	15	1	0	0	0
R7S-South	3° 29´ 48.49˝ S, 37° 18´ 10.96˝ E	47	10	0	0	0	0

### Plant**-**parasitic nematodes identification and counting

After extraction, nematodes suspension was concentrated by removing excess water using a glass pipette, transferred to a counting dish, and adults of the plant**-**parasitic genera were counted using a stereomicroscope. After counting, the nematodes were fixed and subsequently transferred to anhydrous glycerin for mounting on glass slides as described in the study of Singh et al. (2018). The fixed specimens were observed under the microscope, Olympus BX51 DIC Microscope (Olympus Optical, Tokyo, Japan) equipped with an Olympus C5060Wz camera for further analyses.

### Molecular analysis

For molecular analysis, individuals of live nematodes from selected representative PPN populations were first mounted on temporary glass slides to record all necessary morphological and morphometric data by taking pictures and measurements using the above camera**-**equipped microscope. This was followed by DNA extraction from individual nematodes as described in the study of Singh et al. (2018) and the resulting genomic DNA sample was used for the amplification of the partial ITS and D2-D3 region of the 28S of rRNA gene and the *COI* gene of mtDNA. PCR amplification of the partial ITS was done using the primer pair Vrain2F: 5´-CTTTGTACACACCGCCCGTCGCT-3´/Vrain2R: 5´-TTTCACTCGCCGTTACTAAGGGAATC-3´ (Vrain et al., 1992) with thermal profile described in the study of Singh et al. (2019). For amplification of the D2-D3 sequence, the primer pair, 391: 5´-AGCGGAGGAAAAGAAACTAA-3´/501: 5´-TCGGAAGGAACCAGCTACTA-3´ was used as described in the study of Nadler et al. (2006). Partial sequence of *COI* was amplified using the primers, JB3: 5´-TTTTTTGGGCATCCTGAGGTTTAT-3´/JB4.5: 5´-TTTTTTGGGCATCCTGAGGTTTAT-3´ according to Bowles et al. (1992). The PCR products were purified and sent to Macrogen (https://dna.macrogen.com) for sequencing. The obtained sequences were used to make contigs using Geneious 10.0.9 (www.geneious.com) and deposited to GenBank.

### Phylogenetic analysis

Sequences generated in this study were analyzed with other relevant sequences available in GenBank. Multiple alignments of the different DNA sequences were made using MUSCLE with default parameters and followed by manual trimming of the poorly aligned ends using Geneious 10.0.9. Phylogenetic trees were created by using MrBayes 3.2.6 add**-**in of Geneious 10.0.9 with appropriate nucleotide substitution models (see Figs. 4-7) selected using jModelTest 2.1.10. The Markov chains for generating phylogenetic trees were set at 1 × 10^6^ generations, 4 runs, 20% burn**-**in, and subsampling frequency of 500 generations (Huelsenbeck and Ronquist, 2001).

## Results

**Figure 1:**
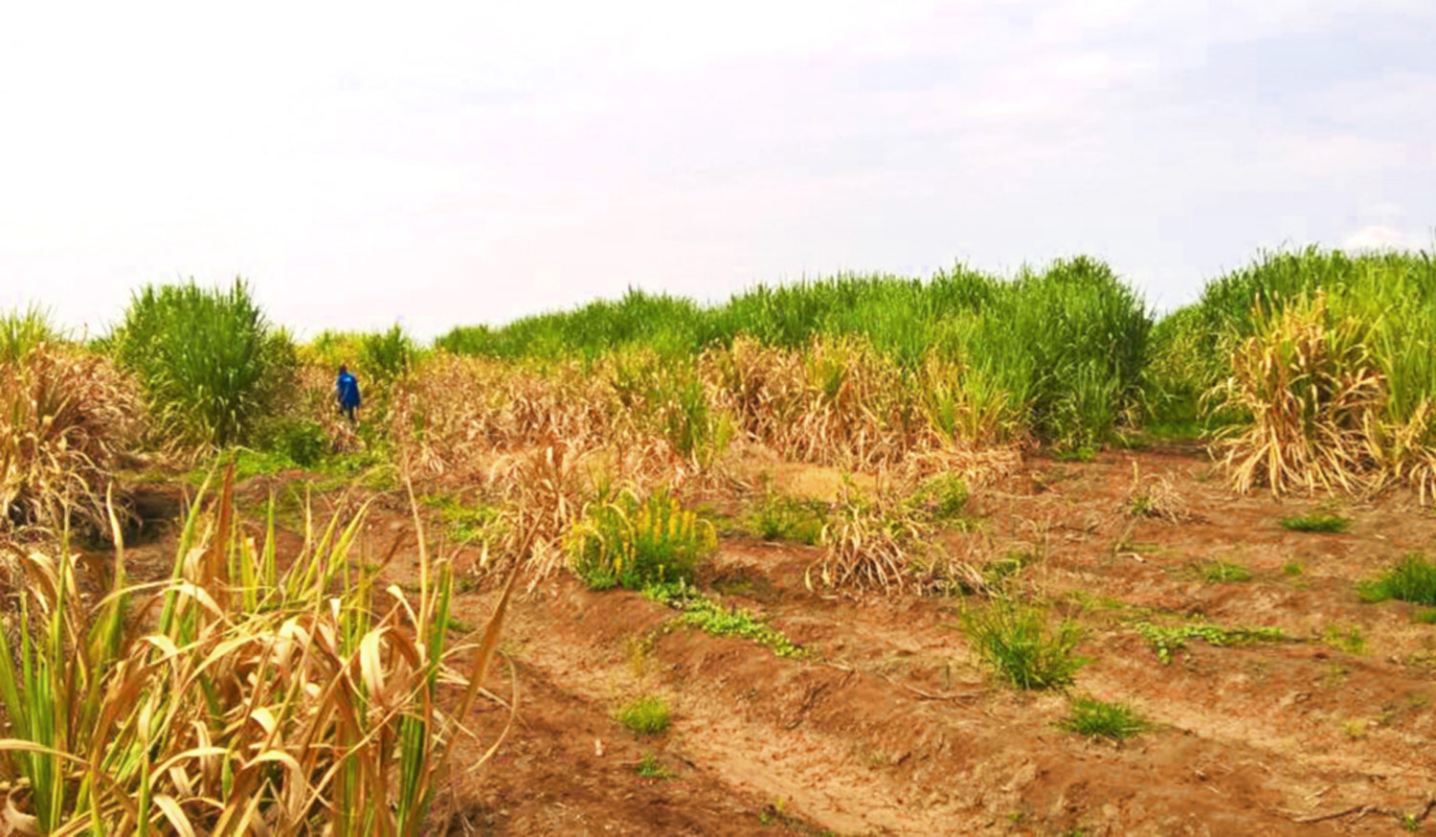
The above-ground view of sugarcane showing stunted growth and yellowing of leaves on the field site F13-South of Tanganyika Planting Company Limited in Kilimanjaro. Soil sample analysis from this field revealed the presence of more than 1,000 immature females and males of *Rotylenchulus parvus* in 100cc of soil.

From the 12 studied sites, six PPN genera were found, i.e. *Helicotylenchus*, *Hemicycliophora*, *Pratylenchus*, *Rotylenchulus*, *Scutellonema*, and *Tylenchorhynchus* (Table 1). *Rotylenchulus* was found to be the most abundant and detected from all the soil samples, with up to 1,000 immature females and males per 100cc of soil. The corresponding sugarcane field (F13-South) with the highest density of *Rotylenchulus* showed above-ground symptoms of stunted sugarcane growth and yellowing of leaves (Fig. 1). The genus *Pratylenchus* was found abundantly in all the sites, except for F10-South, at a density of 2 to 84 nematodes/100cc of soil. The other genera were found in relatively low densities. The genus *Tylenchorhynchus* was found in six samples (1 to 16 nematodes/100cc soil); *Helicotylenchus* in three samples (N54-North, C6-East, and D20-East; 1/100cc soil); *Hemicycliophora* also in three samples (C6-East, D20-East, F10-South; 1-7/100 g soil); and *Scutellonema* was detected from samples N50-North and D20-East (1-5/100cc soil).

A detailed morphological study of the representative populations of the three PPN genera with the highest density, *Rotylenchulus*, *Pratylenchus*, and *Tylenchorhynchus*, revealed the presence of the species *R. parvus* (representative population F13-South), *P. zeae* (E11-East), *T. crassicaudatus*, and *T. ventrosignatus* (F10-South). In this paper, we focus especially on the characterization of *R. parvus* and *T. crassicaudatus*.

**Figure 2:**
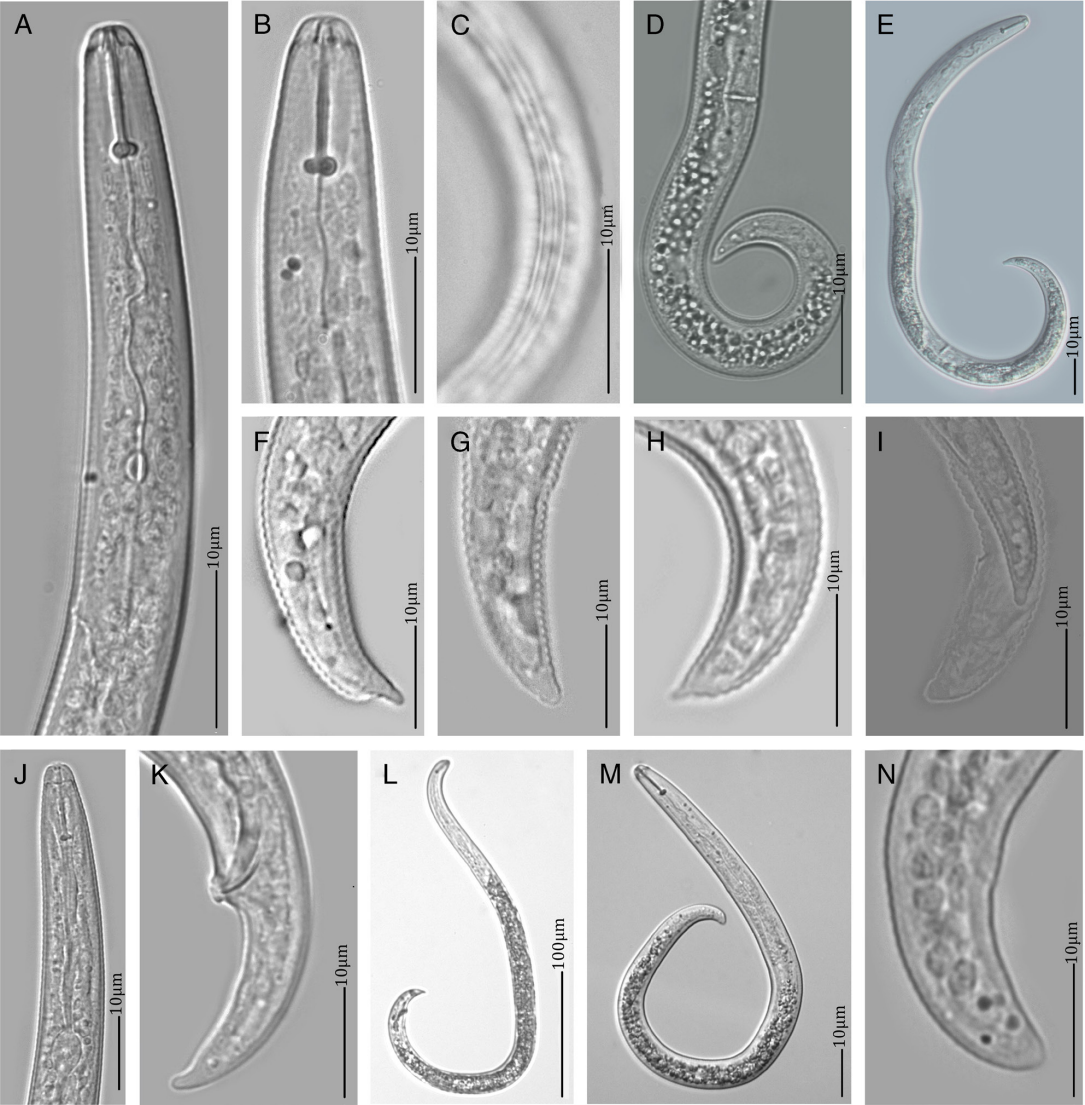
Light microscopy images of *Rotylenchulus parvus* found in the field site F13-South of Tanganyika Planting Company Limited in Kilimanjaro. A-B: Anterior regions of immature females, C: Lateral field showing four distinct incisures, D-E: Female body showing vulval position, F-I: Female tail region showing tail hyaline part, J-K: Anterior and posterior regions of male, respectively, L-M: Whole body of male and juvenile, respectively, N: Tail of juvenile with a rounded tail tip.

*Rotylenchulus parvus* (Williams, 1960a, b) Sher, 1961 (Fig. 2, Tables 2 and 3).

**Table 2.** Morphometrics of immature females and males of *Rotylenchulus parvus* collected from F13-South of Tanganyika Planting Company Limited in Kilimanjaro region of Tanzania.

Character	Immature females	Males
n	28	5
L	327 ± 29 (271-352)	393 ± 38 (342-426)
a	24.9 ± 1.6 (21.9-26.8)	31.1 ± 1.6 (29.4-33.2)
b	3.2 ± 0.5 (2.6-3.9)	3.7 ± 0.2 (3.5-3.9)
c	15.3 ± 0.4 (12.3-17.5)	17.0 ± 0.9 (16.0-18.1)
DGO	17.3 ± 1.1 (16.6-18.0)	–
V	62% (60%-66%)	–
Stylet length	14.5 ± 0.4 (13.1-15.4)	11.9 ± 0.3 (11.7-12.3)
Metenchium length	6.4 ± 0.3 (6.0-6.5)	5.2 ± 0.1 (5.2-5.3)
Telenchium length	8.1 ± 0.2 (6.3-8.7)	6.6 ± 0.2 (6.4-6.9)
Stylet knob width	2.7 ± 0.3 (2.5-2.9)	1.5 ± 0.2 (1.4-1.6)
Stylet knob height	1.6 ± 0.3 (1.4-1.8)	0.9 ± 0.1 (0.9-1.0)
Pharyngeal length	102 ± 8.7 (97.9-110)	109 ± 1.1 (108-110)
SE pore from anterior end	75.4 ± 3.2 (74.0-77.0)	77.9 ± 0.6 (77.5-78.5)
Mid-body diameter	12.7 ± 1.6 (12.7-13.4)	12.6 ± 1.1 (11.2-13.8)
Median bulb length	9.1 ± 0.8 (8.6-9.8)	8.0 ± 0.9 (7.4-8.6)
Median bulb diameter	7.0 ± 0.2 (6.9-7.6)	5.1 ± 0.3 (4.9-5.3)
Lip region diameter	3.8 ± 0.8 (3.1-4.2)	3.5 ± 0.2 (3.4-3.5)
Lip region height	2.4 ± 0.3 (2.2-2.6)	2.1 ± 0.5 (1.6-2.7)
Tail	21.4 ± 0.8 (18.5-25.3)	23.1 ± 2.0 (20.3-25.0)
Hyaline tail (h)	2.5 ± 0.4 (1.3-3.1)	3.4 ± 0.5 (2.9-3.9)
Spicule length	–	16.7 ± 1.2 (16.0-17.5)
Gubernaculum length	–	5.6 ± 0.5 (5.0-6.0)

**Note:** The measurements are given in μm and in the form: mean ± s.d. (range).

**Table 3.** Comparison of important morphological characters and morphometrics of immature females of the Tanzanian *Rotylenchulus parvus* found from sugarcane field F13-South of Tanganyika Planting Company Limited in Kilimanjaro region of Tanzania, with seven other *Rotylenchulus* spp. and the original measurements of *R. parvus* from Mauritius and from South Africa.

Character	*R. clavicaudatus* from South Africa after Van den Berg et al. (2016)	*R. leptus* from South Africa after Van den Berg et al. (2016)	*R. macrodoratus* from Italy after Van den Berg et al. (2016)	*R. macrosoma* from Spain after Castillo et al. (2003a)	*R. macrosomoides* from South Africa after Van den Berg et al. (2016)	*R. parvus (=Helicotylenchus parvus)* from Mauritius after Williams (1960a, b)	*R. parvus* from South Africa after Van den Berg (1978)	*R. parvus* from Kilimanjaro, Tanzania (2017)	*R. reniformis* after Agudelo et al. (2005)	*R. sacchari* from South Africa after Van den Berg et al. (2016)
n	11	14	9	12	10	6	198	28	20	25
Body length	483-624	321-434	407-489	408-510	463-590	210-270	231-432	271-352	340-560	574-796
a	27.6-36.9	25.7-29.9	22.6-27.4	26.3-34.2	31.4-37.3	19.0-24.0	15.3-32.3	21.9-26.8	20.3-31.9	28.8-36.9
b	3.8-4.7	2.9-4.3	2.9-4.0	3.5-4.4	3.8-4.7	2.9-3.3	2.0-4.4	2.6-3.9	2.3-4.1	3.1-5.0
c	9.1-11.8	14.5-17.3	18.5-22.2	11.7-16.8	14.1-21.4	16.0-20.0	13.1-27.1	12.3-17.5	10.0-22.8	26.1-43.0
DGO	11.0-18.0	20.0-24.5	13.0-18.0	22.0-27.0	30.0-33.0	–	7.7-17.3	16.6-18.0	–	8.0-15.5
V%	55-61	59-65	62-67	59-64	76-83	61-65	56-69	60-66	66-73	62-71
Stylet length	16.0-20.0	12.5-14.5	21.0-24.0	15.0-18.0	21.5-25.5	ca 12.5	10.7-19.9	13.1-15.4	16.0-22.0	26.5-34.5
Tail length	43.0-66.0	20.0-28.0	20.0-24.0	26.0-40	26.5-36.0	–	10.5-27.2	18.5-25.3	–	15.5-28.0
Hyaline length	19.0-35.5	1.4-4.0	8.0-12.0	9.0-12.0	17.0-25.5	–	0.8-5.2	1.3-3.1	4.0-9.0	4.5-10.5
Tail shape and structure	Broadly rounded, clavate, annulated tip	Tapering gradually to a finely rounded annulated tip	Bluntly rounded and slightly annulated terminus	Bluntly rounded and prominently annulated terminus	Tapering to a rounded or slightly clavate annulated tip	Arcuate, conoid, terminus knob like, length twice anal body diameter	Conoid, ventrally arcuate with sharply pointed or irregular or broadly rounded tip	Conoid, tapering to rounded tip	Tapering to rounded terminus	Broadly rounded, faint annulation at the tip
Habitus	Open letter C to complete circle	Open Figure 6 to 1.5 circle	Closed C-shape	Closed C-shape	C to curved into 1.5 circles	Loose spiral resembling 6-shape	C- or 6-shape or complete circle	C- to 6-shape	Open spiral to C-shape	Almost straight to C-shape rarely 1.5 circles
Labial region	Slightly sloping anteriorly to a slightly rounded tip, not set off	Sloping to a flattened tip	Conoid rounded, not set off	Conoid rounded, not set off	High, sloping slightly to a slightly flat or rounded tip	Continuous with neck, sloping to a rounded, distinctly flattened front	Broadly rounded, almost flat, not set off	Low, not set off and flattened front	High, conoid and continuous	Slightly sloping to flattened tip
Labial annuli	Not present	4 to 5	Fine annuli	Fine annuli	Not visible	–	3-5 faint annuli	4 to 5 faint annuli	4 to 6	5 to 6 faint annuli

**Note:** Lengths are given in μm and measurement are presented in a range.

### Description

#### Immature female

Females are vermiform. Heat relaxed specimens curve ventrally in the form of the letter C or Figure 6. Lip region is conoid, flattened, sometimes slightly rounded, not set off and with 4 to 5 fine indistinct annuli. Labial framework is well-developed and stylet is sclerotized with rounded knob sloping posteriorly. Length of telenchium is always about 2 μm longer than that of metenchium. Dorsal pharyngeal gland opening is about 17 μm from the stylet base. Median bulb is strong, slightly longer than its width and with distinct valves. Pharyngeal gland overlaps the intestine ventrally, more predominantly on lateral side. Secretory-excretory pore is about 75 μm from the anterior end. Vulva is situated postmedially in about 60 to 66% of the body length from anterior end. Reproductive system is didelphic amphidelphic with two outstretched genital tracts and reflexed ovarian tips. Lateral field is with four distinct incisures and three equal bands. Tail is with 18 to 22 annuli, tapering to a rounded, coarsely annulated tip, about 21 μm long. Length of hyaline tail part is fairly short, between 1.3 and 3.1 μm long. Phasmid is pore like, located halfway of tail.

#### Males

Males are rare to be found. Heat relaxed specimens curves ventrally from C- to 6-shapes. Body of males is slightly longer and slender than that of females. Anterior part is less developed, with shorter and weaker stylet and knobs compared to that of females. Secretory-excretory pore is at about the same level from anterior end as in immature females. Lateral field is with four incisures and three distinct bands. Tail and hyaline tail part is slightly longer than that of immature females. Spicules are arcuate ventrally, about 17 μm long with linear gubernaculum about 6 μm long.

### Molecular characterization

#### ITS of rDNA

Five ITS sequences (MK558212 to MK558216) with intraspecific variation of up to 0.3% (1-2 bp) were generated for the Tanzanian *R*. *parvus*, and the resulting alignment, which included 79 other ITS sequences from seven *Rotylenchulus* species and South African *R. parvus*, was 361 bp long. The phylogenetic tree (Fig. 4) inferred revealed a sister relationship of the *R. parvus* from Tanzania and South Africa (PP = 0.97). However, remarkably, the Tanzanian *R*. *parvus* sequences were 65 to 77 bp (18-21%) different from that of the South African *R*. *parvus* (KT003771 to KT003779). The phylogenetic position of *R. parvus* in respect to other *Rotylenchulus* species remains unresolved in the ITS tree.

#### D2-D3 of 28S rDNA

Four D2-D3 sequences (MK558202 to MK558205) with intraspecific variation of up to 0.3% (2-4 bp) were obtained. The D2-D3 alignment was 611 bp long and included 85 other sequences from eight known and four unknown *Rotylenchulus* species. The D2-D3 sequences were found to be 74-80 bp (12-13%) different from the sequences of South African *R. parvus* (KT003734 to KT003738) and did not form a clade with them in the D2-D3 tree. They formed a maximally supported clade with an unidentified *Rotylenchulus* sp. from Indonesia (unplubl. sequence; about 20 bp differences) and with another unidentified *Rotylenchulus* sp. from the USA (MF425701; 3.0-3.7% or 18-19 bp difference). The later *Rotylenchulus* sp. was found to fit morphologically *R. parvus* (Subbotin et al., 2017). This clade has a poorly supported relation with *R. sacchari* (PP = 0.64) (Fig. 5). *Rotylenchulus parvus* from South Africa formed a well-supported clade with *R*. *clavicaudatus* and some unidentified *Rotylenchulus* species (PP = 0.98).

#### *COI* of mtDNA

Three 100% similar *COI* sequences (MK558209 to MK558211) from the Tanzanian *R*. *parvus* were aligned (393 bp) with 49 other *COI* sequences from six known and six unknown *Rotylenchulus* species. The Tanzanian *R*. *parvus* sequences were found to be 50 to 55 bp (13-14%) different from that of the South African *R*. *parvus* (KT003732) and both the *R*. *parvus* populations were in a well-supported clade (PP = 0.98) together with six other unidentified *Rotylenchulus* sequences from South Africa (Fig. 6). The sequences of *R. parvus* and the unidentified *Rotylenchulus* spp. sequences of South Africa appeared to have a sister relationship with *R. macrodoratus* (PP = 0.86).

#### Remarks

The morphology and morphometrics of the current immature female specimens correspond very well with that of the original description of *R*. *parvus* (=*Helicotylenchus parvus*), found also around sugarcane roots in Mauritius by Williams (1960a, b). *Rotylenchulus parvus*, in general, compared to other related species, has a slightly smaller body, 3 to 5 labial annuli visible under the light microscope, and a relatively short tail hyaline part (0.8-5.2 μm). However, in the original description, the information on the number of labial annuli and hyaline tail length was not given, and mature females and males were also not reported. The Tanzanian *R. parvus* is also similar to a South African *R. parvus* population detected around maize roots by Van den Berg (1978) and to a population found in soil of a cotton field in Greenhouse in California by Dasgupta et al. (1968). However, some minor differences between our Tanzanian *R. parvus* and the South African *R. parvus* can be observed, i.e. hyaline tail length (1.3-3.1 vs 0.8-5.2 μm), stylet length (13-15 vs 11-20 μm), and the number of labial annuli (4-5 vs 3-5) (Table 3). Mature females could not be compared as no mature females were found in current study. Van den Berg et al. (2016) provided the first molecular data of this species including the ITS, D2-D3, *COI*, and *hsp90* sequences for another South African *R. parvus* population of only matured obese females. Remarkably, our molecular data were found to be considerably different from this population. Nevertheless, the close relatedness between our Tanzanian and this South African population is indicated by a sister relationship based on both COI and ITS phylogenetic analyses. However, the D2-D3 tree indicated *R. parvus* as a paraphyletic species with *R. sacchari* and *R. clavicaudatus* diverging independently. The phylogenetic relations of *R. parvus* with *R. sacchari* and *R. clavicaudatus* are, however, not well supported in the D2-D3 tree.

Both the South African and the Tanzanian *R. parvus* populations are in similar geographic proximity of the type location, whereas the latter was found from the same crop (sugar cane) as was originally described, which indicates that it is slightly more likely that our Tanzanian population represents the type species. However, without the nematode sequences from the type location, it cannot be concluded whether either of the two sequences represent the genuine *R. parvus.* An alternative explanation to the high sequence variations in *R. parvus* could be the existence of two distinct types of rRNA operons as was found in at least three other *Rotylenchulus* spp. (Nyaku, Kantety, Tilahun, Lawrence, Soliman, Cebert, and Sharma 2013; Nyaku, Sripathi, Kantety, Gu, Lawrence and Sharma, 2013; Van den Berg et al., 2016). However, this hypothesis is not supported by the independent COI analyses, unless a remarkably high intraspecific COI variability or the presence of two distinct mitochondrial genomes within *R. parvus* is also assumed. This can only be clarified by genomic studies for *R. parvus* and other *Rotylenchulus* spp. A voucher slide containing three immature females and one male (UGnem-213) was deposited at Nematology Research Unit, UGent.

**Figure 3:**
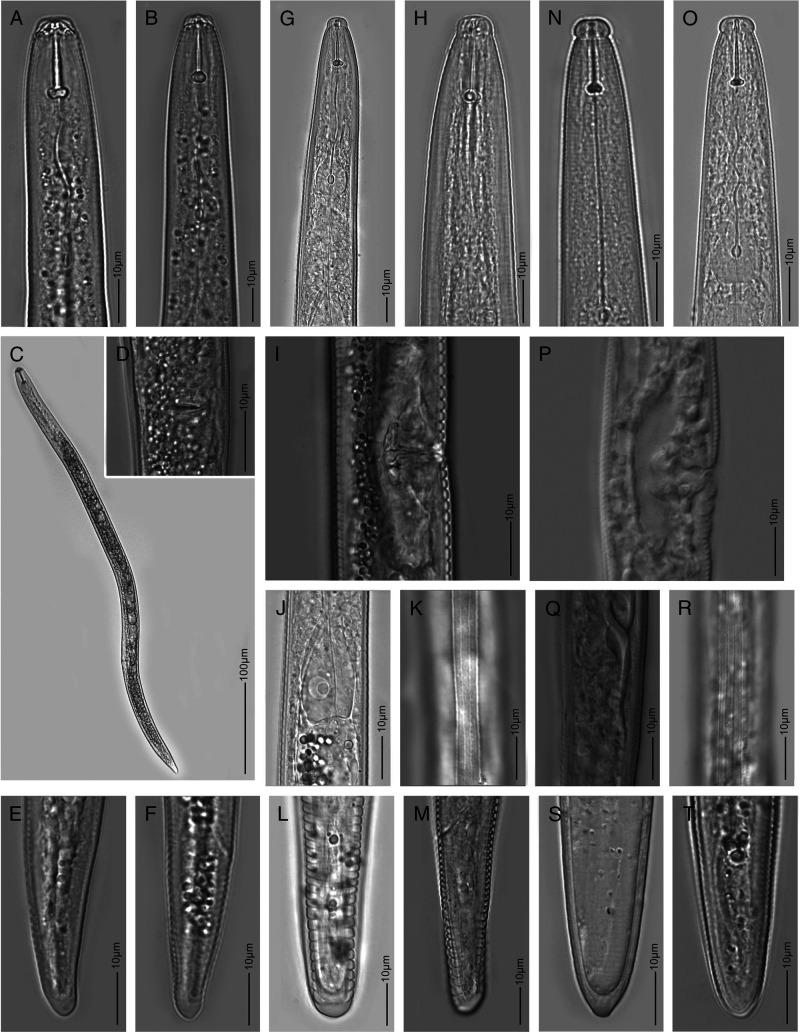
Light microscopy images of three plant parasitic nematode species detected in this study. A-F: *Pratylenchus zeae,* G-M: *Tylenchorhynchus crassicaudatus*, and N-T: *Tylenchorhynchus ventrosignatus*.

*Tylenchorhynchus crassicaudatus* (Williams, 1960a, b) (Fig. 3)

**Figure 4:**
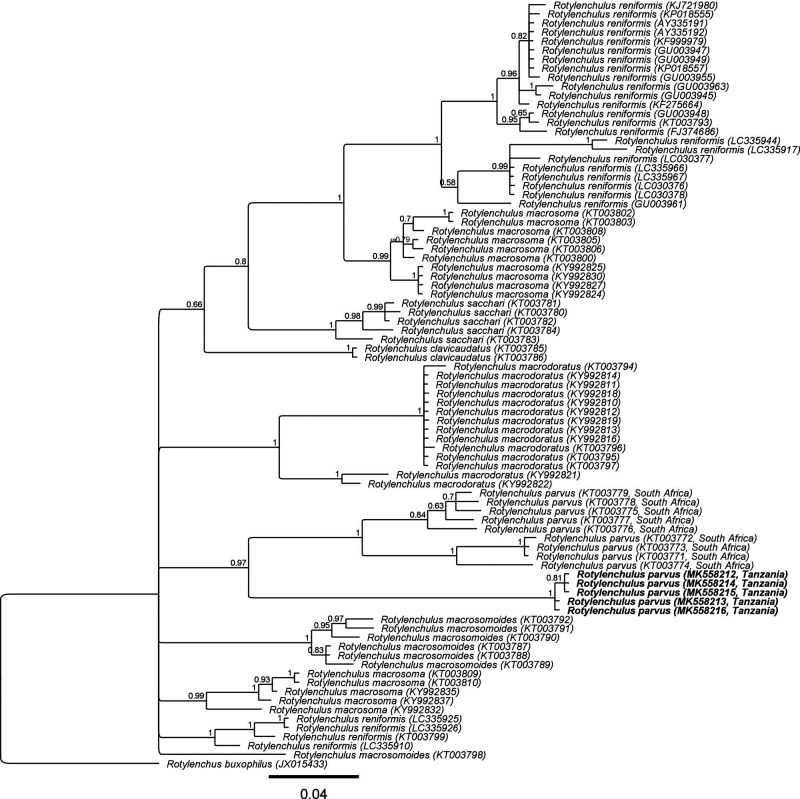
Phylogenetic relationships of *Rotylenchulus parvus* from Tanzania with seven other *Rotylenchulus* species. Bayesian 50% majority rule consensus tree as inferred from the analysis of ITS of rDNA sequences under GTR + I + G model. Posterior probabilities of more than 0.5 are given for appropriate clades.

**Figure 5:**
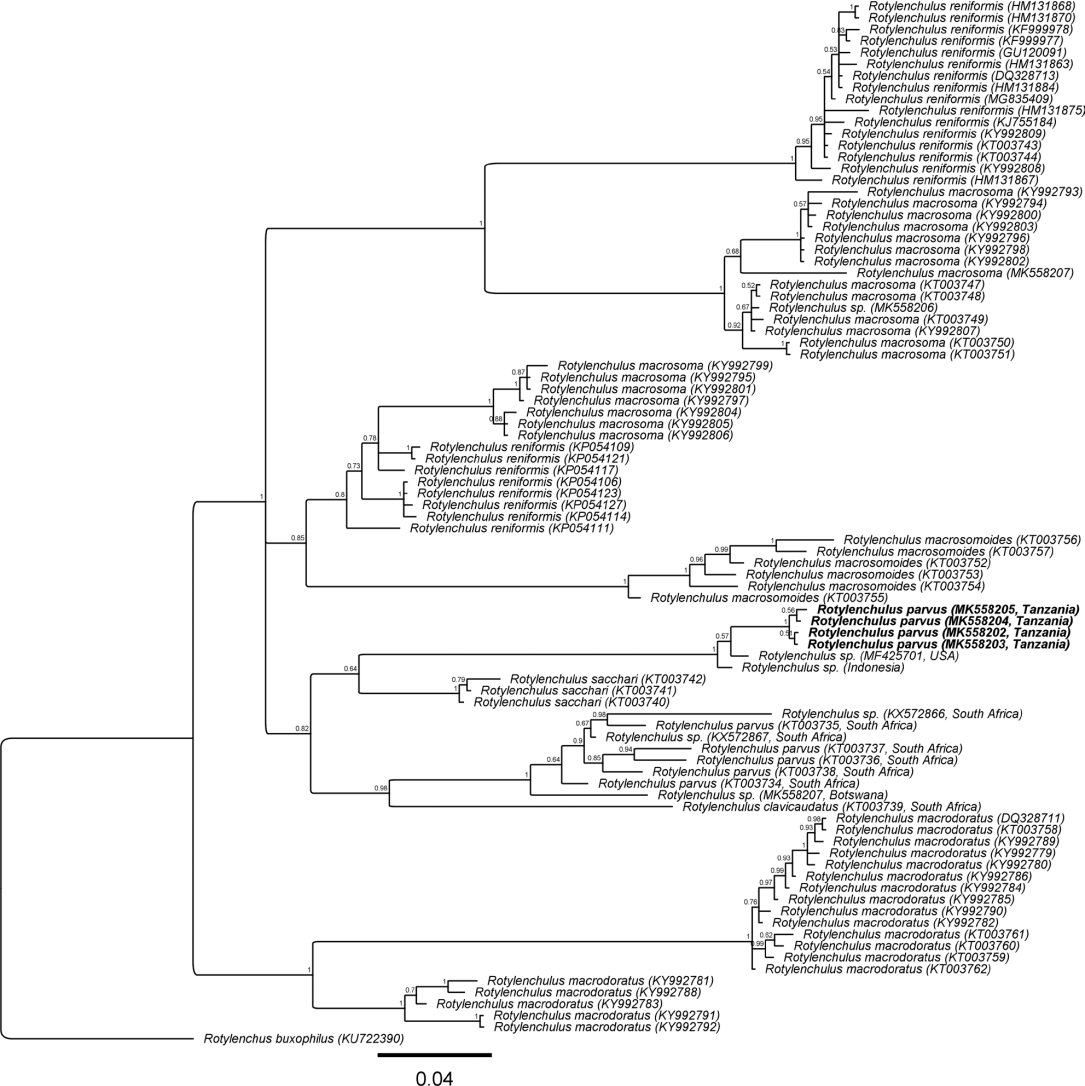
Phylogenetic relationships of the Tanzanian *Rotylenchulus parvus* with eight known and four unknown *Rotylenchulus* species. Bayesian 50% majority rule consensus tree as inferred from the analysis of D2-D3 of 28S rDNA sequences under GTR + I + G model. Posterior probabilities of more than 0.5 are given for appropriate clades.

**Figure 6:**
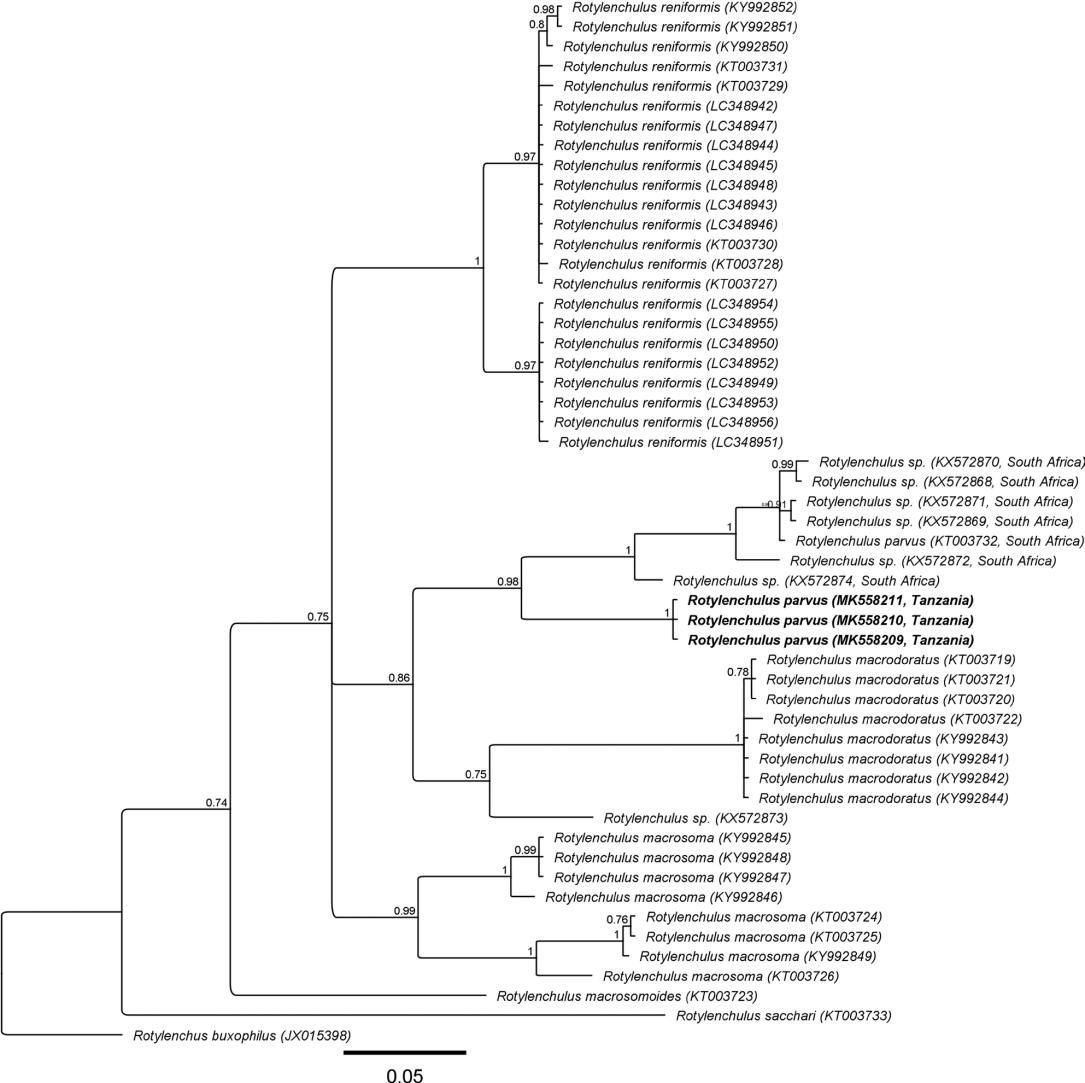
Phylogenetic relationships of *Rotylenchulus parvus* from Tanzania with six known and six unknown *Rotylenchulus* species. Bayesian 50% majority rule consensus tree as inferred from the analysis of COI of mtDNA sequences under GTR + I + G model. Posterior probabilities more than 0.5 are given for appropriate clades.

The morphology and morphometrics (Table 4) of *T. crassicaudatus* females (*n* = 5) were in agreement with the original description of the species by Williams (1960a, b) except for slightly longer body (725-755 vs 580-690 μm) and a more sub-cylindrical tail shape with 21-26 tail annuli vs clavate tail with 17 to 19 tail annuli in the original description. No males were found in this study. This population was also similar to a population of *T. crassicaudatus* from Niger with a clear absence of a post-anal intestinal sac and variable tail shape, from subcylindrical to clavate, and also similar to another *Tylenchorhynchus* population from a rice field in Haiti that fits more to the descriptions of *T. agri* (Ferris, 1963) (subcylindrical tail shape, females with post-anal intestinal sac, and morphometrics) (unpubl. data; Table 4). However, in this population from Haiti, female individuals without post-anal intestinal sac were also present.

**Table 4.** Comparison of important morphological characters and morphometrics of the Tanzanian *Tylenchorhynchus crassicaudatus*, found from sugarcane field F10-South of Tanganyika Planting Company Limited in Kilimanjaro region of Tanzania, with the original measurements of *T. crassicaudatus* from Mauritius and *T. agri* from the USA along with three other populations of *T. agri* and *T. crassicaudatus* from Niger, USA and Haiti.

Character	*T. crassicaudatus* from cane roots in Mauritius (Williams, 1960a, b)	*T. agri* from corn field in Urbana, Illinois, USA (Ferris, 1963)	*T. crassicaudatus* from Niger (2011)	*T. agri* from dwarf date palm in Lake Worth, Florida, USA (Handoo et al., 2014)	*T. crassicaudatus* from sugarcane field in Kilimanjaro, Tanzania (2017)	*T. agri* from rice field in Haiti (2018)
n	5	10	6	1	5	10
Body length	580-690	660-770	521-666	662	725-755	538-695
a	28-33	28-33	27-31	32.9	28-35	23-32
b	4.9-5.7	4.7-5.5	4.3-5.0	4.9	4.7-5.2	4.4-5.3
c	14-15	15-21	13-16	13.3	14-17	12-15
V%	53-57	55-58	53-59	55.1	53-55	51-55
Stylet length	ca 20	20-23	18-20	21	19-20	18-19
Tail length	ca 50	–	37-42	50	43-56	43-53
Tail annuli number	17-19	18-26	17-23	25	21-26	18-20
Tail shape	Straight to slightly arcuate, thick and clavate	Subcylindrical	Clavate to subcylindrical	Subcylindrical	Subcylindrical	Subcylindrical
Tail terminus	Smooth, rounded and virtually hemispherical	Broadly rounded and smooth	Smooth, rounded and virtually hemispherical	Smooth and broadly rounded	Smooth and rounded	Broadly rounded and smooth
Post anal intestinal sac	Not mentioned (absent?)	Present	Absent	Not mentioned	Not present	Present or absent
Labial region	Broadly rounded, low, not setoff	Separated from body contour by slight depression	Broadly rounded, low, not set off, sometimes seems separated from body contour by slight depression	Continuous with body contour or separated by slight depression	Broadly rounded, not set off, sometimes seems separated from body contour by slight depression	Seems separated from body contour by slight depression
Labial annuli	3	3 + labail disc	3	3 to 4	3	3

**Note:** Lengths are given in μm and measurement are presented in range.

### Molecular characterization

**Figure 7:**
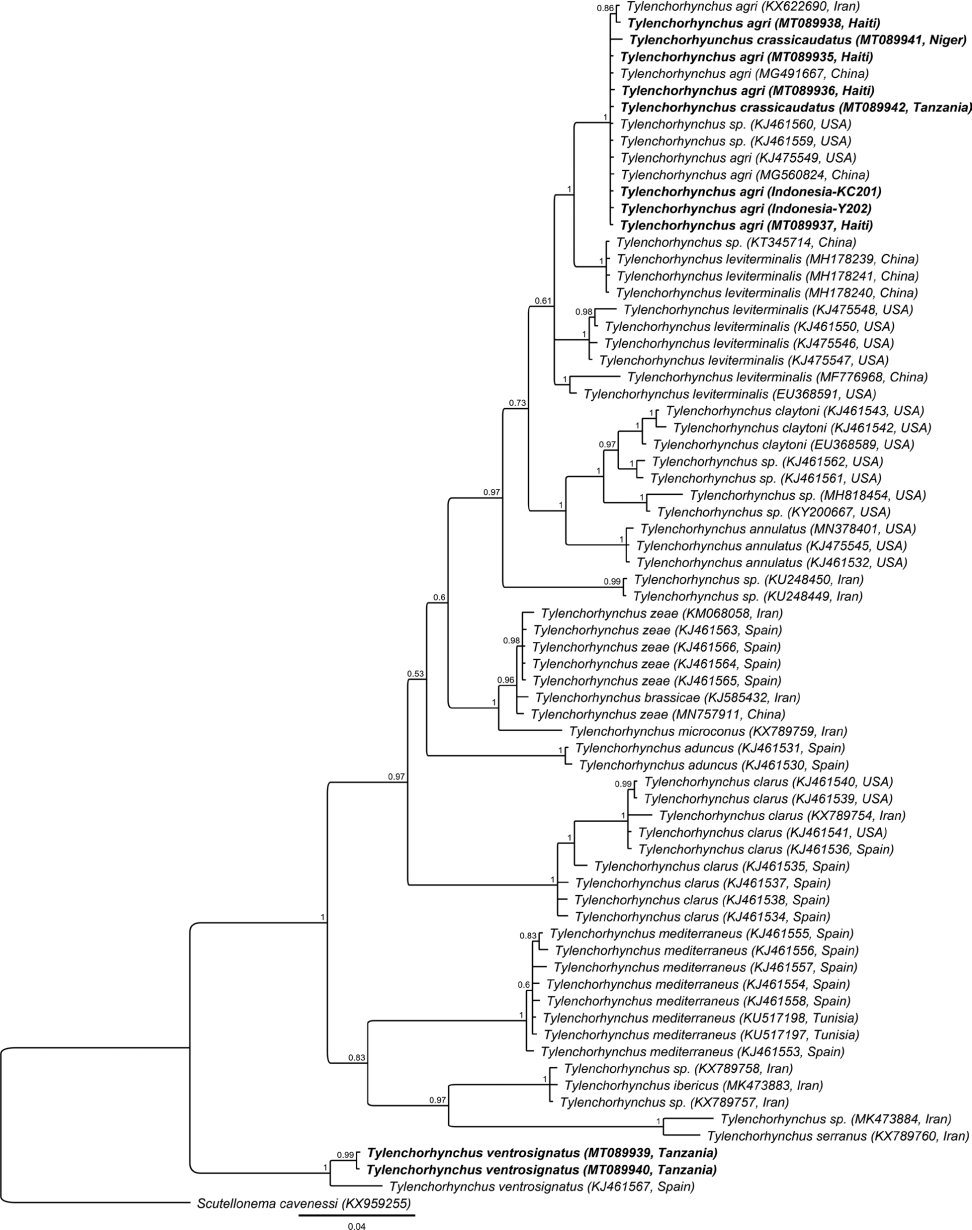
Phylogenetic relationships of *Tylenchorhynchus crassicaudatus* and *Tylenchorhynchus ventrosignatus* from Tanzania with other *Tylenchorhynchus* species. Bayesian 50% majority rule consensus tree was inferred from the analysis of D2-D3 of 28S rDNA sequences under GTR + G model. Posterior probabilities more than 0.5 are given for appropriate clades.

The D2-D3 sequence (MT089942) from our Tanzanian *T. crassicaudatus* population was 100% identical to the sequences of *T. agri* from China (MG491667 and MG560824), Iran (KX622690), the USA (KJ475549, KJ475559, and KJ475560), Haiti (MT089935 to MT089938), and Indonesia (Lestari et al., in prep), and only two out of 690 bp different from a *T. crassicaudatus* sequence from Niger (MT089941). In the D2-D3 tree (Fig. 7), the *T. agri* and *T. crassicaudatus* sequences formed a maximally supported clade without internal resolution. Also, the 18S sequences (MT076074) from *T. crassicaudatus* from Niger and *T. agri* from Haiti (MT076072 and MT076073) were virtually identical (one out of 880 bp different).

### Synonymisation of *T. agri* syn. n. with *T. crassicaudatus*

*Tylenchorhynchus agri* has been differentiated from *T. crassicaudatus* by a more sub-cylindrical tail shape vs a more clavate tail and the presence vs absence of a post-anal intestinal sac/extension. Following the table of Handoo (2000), *T. agri* differs from *T. crassicaudatus* based on lip region (offset vs continuous), lip annuli number (four vs three), tail shape (sub-cylindrical vs clavate), tail tip annulation (smooth vs annulated), and other minor morphometrical differences (see Table 4). However, in this table, the lip annuli number of *T. agri* (four instead of three) and the tail tip annulation of *T. crassicaudatus* (annulated instead of smooth) have been reported incorrectly. Furthermore, the lip region of both species appears to be similar based on their originals descriptions. In the dichotomous *Tylenchorhynchus* key by Geraert (2011), the two species have been differentiated solely by the presence vs absence of the post-anal intestinal sac.

Based on our study in combination with the available literature, we can summarize the following elements: (1) even within a single population, *T. crassicaudatus* shows clear morphological variations, for example, the presence of both sub-cylindrical and clavate tail in the Niger population; (2) a population close to *T. crassicaudatus* (Niger) and a population more close to *T. agri* (Haiti) are molecularly virtually identical based on both D2-D3 and the 18S sequences; and (3) global-wide sampling shows that the D2-D3 sequences of all investigated *T. crassicaudatus*/*T. agri* populations are virtually identical and are all within a well-supported clade.

Hence, the differential morphological traits of these two species appear to be morphological variations of *T. crassicaudatus*, a globally distributed species. Although sequences from the type location material are needed to assure that the two species are synonymous, a comparison of the original descriptions and a comprehensive morphological and molecular analyses of populations from different part of the world suggests that *T. agri* syn. n. should be considered as a junior synonym of *T*. *crassicaudatus.*

*Tylenchorhynchus ventrosignatus* Tobar Jiménez, 1969 (Fig. 3)

Only female individuals of the species were found in the representative population, and their morphology and morphometrics (n = 10) were in agreement with the original description from Tobar Jiménez (1969) except for slightly longer stylets (11-15 vs 14-21 μm) and longer tails (36-43 vs 32-56 μm). Two identical D2-D3 sequences were generated (MT089939 and MT089940), which were six out of 724 bp different from a sequence of *T. ventrosignatus* from Spain (KJ461567). Six partial ITS sequences of about 960 bp (MT089943 to MT089948) showed an intraspecific sequence variation of 2 to 6 bp and appeared to be 36 to 45 bp different from a *T. ventrosignatus* sequence from Spain (KJ461596). However, despite the sequence differences, the Tanzanian *T. ventrosignatus* sequences formed a maximally supported clade together with the Spanish sequence in both the D2-D3 tree (Fig. 7) and the ITS tree (tree not shown). A slide containing three females was deposited at Nematology Research Unit, Ugent (UGnem-215) as voucher material.

*Pratylenchus zeae* Graham, 1951 (Fig. 3)

The morphology and morphometrics of females (n = 4) from our representative population agree with the original description of *P. zeae* by Graham (1951). Three 100% similar partial *COI* sequences of about 400 bp (MT076075 to MT076077) of *P. zeae* were produced in this study, and they were 1 to 2 bp different from the existing *P. zeae* sequences from China (KY424055 to KY424063) and the USA (KU522440). Our sequences are in a maximally supported clade with other available *P. zeae* sequences (data not shown). Four females in one slide (UGnem-211) was kept as voucher material at the Nematology Research Unit, UGent.

## Discussion

The impact of PPN on sugarcane cultivation and how to reduce the associated damage is widely studied (Cadet and Spaull, 2003; Blair and Stirling, 2007; Omarjee et al., 2008; Sikora et al., 2018). However, PPN may still be overlooked due to the lack of expertise and diverted attention to more visible pests such as insects and fungi. Although sugarcane is an important cash crop in Tanzania, an investigation on the presence of PPN has not yet been done. This study revealed, for the first time, the presence of several parasitic nematodes in sugarcane fields at the TPC Limited in Kilimanjaro region. *Rotylenchulus* and *Pratylenchus* have been found in high density in virtually all of the analyzed soil samples and also in some root samples (although nematodes were not counted), and they pose a potential threat to the sugarcane production of Tanzania. The most important species identified in this study, *R. parvus* and *P. zeae*, are already well known as notorious pests causing yield reduction of sugarcane plantations (Stirling and Blair, 2001; Cadet and Spaull, 2003). Remarkably, the observed high density of *R*. *parvus* in one of the fields coincided with above-ground symptoms, including stunted sugarcane growth and yellowing of leaves and root necrosis. However, as this field was also found associated with the white grubs of the insect pest *Cochliotis melolonthoides*, the relation and quantification of the nematodes vs the white grubs effects, alone and in combination, to the sugarcane production need to be further investigated.

Our study confirms that cryptic species represent an important component of biodiversity, also in plant-parasitic nematodes (Palomares-Rius et al., 2014) and molecular techniques may be the only practical approach to recognize them (Powers, 2004). DNA barcoding is known to be a reliable diagnostic strategy, although only valid with a clear link between DNA sequences and the morphospecies (Janssen, Karssen, Couvreur, Waeyenberge and Bert, 2017). However, collecting topotype material is often the only option in order to link formerly described morphospecies to DNA barcodes (Janssen, Karssen, Orlando, Subbotin and Bert, 2017). This is especially the case for cryptic species in order to know which of the representative sequences represents the genuine morphospecies.

Nevertheless, despite the taxonomical problems associated with cryptic species, this type of study of uncovering the PPN associated with sugarcane in Tanzania is the first step to facilitate the development of appropriate management strategies to minimize damage by PPN associated with sugarcane in Tanzania and sugarcane in general.

## References

[ref001] Agudelo P., Robbins R. T., Stewart J. M. and Szalanski A. L. 2005 Intraspecific variability of *Rotylenchulus reniformis* from cotton-growing regions in the United States. Journal of Nematology 37:105–114. 19262849 PMC2620946

[ref002] Arndt C., Pauw K. and Thurlow J. 2010 Biofuels and economic development in Tanzania (No. 966) International Food Policy Research Institute (IFPRI), Washington.

[ref003] Berry S. D., Cadet P. and Spaull V. W. 2017 “Nematode pests of sugarcane”, in Fourie H., Spaull V. W., Jones R. K., Daneel M. S. and De Waele D. (Ed.), Nematology in South Africa: a view from the 21st century Springer, Cham, 261–284.

[ref004] Berry S. D., Fargette M., Spaull V. W., Morand S. and Cadet P. 2008 Detection and quantification of root-knot nematode (*Meloidogyne javanica*), lesion nematode (*Pratylenchus zeae*) and dagger nematode (*Xiphinema elongatum*) parasites of sugarcane using real-time PCR. Molecular and Cellular Probes 22:168–176. 18378423 10.1016/j.mcp.2008.01.003

[ref005] Blair B. L. and Stirling G. R. 2007 The role of plant-parasitic nematodes in reducing yield of sugarcane in fine-textured soils in Queensland, Australia. Australian Journal of Experimental Agriculture 47:620–634.

[ref006] Bowles J., Blair D. and McManus D. P. 1992 Genetic variants within the genus *Echinococcus* identified by mitochondrial DNA sequencing. Molecular and Biochemical Parasitology 54:165–173. 1435857 10.1016/0166-6851(92)90109-w

[ref007] Cadet P. and Spaull V. W. 2003 Effect of nematodes on the sustained production of sugarcane in South Africa. Field Crops Research 83:91–100.

[ref008] Castillo P., Vovlas N., Subbotin S. and Troccoli A. 2003a A new root-knot nematode, *Meloidogyne baetica* n. sp. (Nematoda: Heteroderidae), parasitizing wild olive in Southern Spain. Phytopathology 93:1093–1102. 18944092 10.1094/PHYTO.2003.93.9.1093

[ref009] Chirchir A. K., Kimenju J. W., Olubayo F. M. and Mutua G. K. 2008 Abundance and distribution of plant parasitic nematodes associated with sugarcane in Western Kenya. Asian Journal of Plant Pathology 2:48–53.

[ref010] Cock M. J. and Allard G. B. 2013 Observations on white grubs affecting sugar cane at the Juba Sugar Project, South-Western Somalia, in the 1980s, and implications for their management. Insects 4:241–272. 26464389 10.3390/insects4020241 PMC4553522

[ref011] Dasgupta D. R., Raski D. J. and Sher S. A. 1968 A Revision of the Genus *Rotylenchulus* Linford and Oliveira, 1940 (Nematoda: Tylenchiclae). Proceedings of the Helminthological Society of Washington 35:169–192.

[ref012] Geraert E. 2011 The Dolichodoridae of the world: identification of the family Dolichodoridae Academia Press, Ghent.

[ref013] Graham T. W. 1951 Nematode root rot of tobacco and other plants. Bulletin of the South Carolina Experiment Station 390:1–125.

[ref014] Greathead D. J. 1970 White sugar-cane scales (*Aulacaspis* spp.) (Diaspididae: Hemiptera) in East Africa with notes on their natural enemies. East African Agricultural and Forestry Journal 36:70–76.

[ref015] Ferris V. R. 1963 *Tylenchorhynchus silvaticus* n. sp. and *Tylenchorhynchus agri* n. sp. (Nematoda: Tylenchida). Proceedings of the Helminthological Society of Washington 30:165–168.

[ref016] Handoo Z. A. 2000 A key and diagnostic compendium to the species of the genus *Tylenchorhynchus* Cobb, 1913 (Nematoda: Belonolaimidae). Journal of Nematology 32:20–34. 19270946 PMC2620427

[ref017] Handoo Z. A., Palomares-Rius J. E., Cantalapiedra-Navarrete C., Liébanas G., Subbotin S. A. and Castillo P. 2014 Integrative taxonomy of the stunt nematodes of the genera *Bitylenchus* and *Tylenchorhynchus* (Nematoda, Telotylenchidae) with description of two new species and a molecular phylogeny. Zoological Journal of the Linnean Society 172:231–264.

[ref018] Huelsenbeck J. P. and Ronquist F. 2001 MRBAYES: Bayesian inference of phylogenetic trees. Bioinformatics 17:754–755. 11524383 10.1093/bioinformatics/17.8.754

[ref019] Janssen T., Karssen G., Couvreur M., Waeyenberge L. and Bert W. 2017 The pitfalls of molecular species identification: a case study within the genus *Pratylenchus* (Nematoda: Pratylenchidae). Nematology 19:1179–1199.

[ref020] Janssen T., Karssen G., Orlando V., Subbotin S. and Bert W. 2017 Molecular characterization and species delimiting of plant parasitic nematode of the genus *Pratylenchu*s from the Penetrans group (Nematoda: Pratylenchidae). Molecular Phylogenetics and Evolution 117:30–48. 28778818 10.1016/j.ympev.2017.07.027

[ref021] Jepson W. F. 1956 The biology and control of the sugar-cane chafer beetles in Tanganyika. Bulletin of Entomological Research 47:377–397.

[ref022] Katundu J. M. and Ramadhani T. 1988 Integrated control of sugarcane white scale in Tanzania. Taro Newsletter 3:15–19.

[ref023] Msechu Z. E. and Keswani C. L. 1978 Status of Sugarcane Smut (Ustilago Scitaminea Sydow) in Tanzania. East African Agricultural and Forestry Journal 44:164–170.

[ref024] Nadler S. A., Bolotin E. and Stock S. P. 2006 Phylogenetic relationships of *Steinernem*a Travassos, 1927 (Nematoda: Cephalobina: Steinernematidae) based on nuclear, mitochondrial and morphological data. Systematic Parasitology 63:159–179. 10.1007/s11230-005-9009-3 16541298

[ref025] Noronha M. D. A., Muniz M. D. F. S., Cruz M. D. M., Assunção M. C., Castro J. M. D. C., Oliveira E. R. L. D., Miranda C. G. D. S. and Machado A. C. Z. 2017 *Meloidogyn*e and *Pratylenchus* species in sugarcane fields in the state of Alagoas, Brazil. Ciência Rural 47, 3pp.

[ref026] Nyaku S. T., Sripathi V. R., Kantety R. V., Gu Y. Q., Lawrence K. and Sharma G. C. 2013 Characterization of the two intra-individual sequence variants in the 18S rRNA gene in the plant parasitic nematode, *Rotylenchulus reniformis* . PLoS ONE 8:e60891. 23593343 10.1371/journal.pone.0060891 PMC3623918

[ref027] Nyaku S. T., Kantety R. V., Tilahun Y., Lawrence K. S., Soliman K. M., Cebert E. and Sharma G. C. 2013 18S and ITS1 genomic sequence variations in *Rotylenchulus reniformi*s isolates from Alabama. The Journal of Cotton Science 17:184–194.

[ref028] Omarjee J., Balandreau J., Spaull V. W. and Cadet P. 2008 Relationships between *Burkholderia* populations and plant parasitic nematodes in sugarcane. Applied Soil Ecology 39:1–14.

[ref029] Palomares-Rius J. E., Cantalapiedra-Navarrete C. and Castillo P. 2014 Cryptic species in plant-parasitic nematodes. Nematology 16:1105–1118.

[ref030] Paray N. B., Mmanga S., Hatting J. L., Conlong D. E. and Ganeshan S. 2012 Detection, isolation and characterisation of white grub (Coleoptera: Scarabaeidae) pathogens in Mauritius and Tanzania. Proceedings of the Annual Congress-South African Sugar Technologists’ Association 85:123–128.

[ref031] Powers T. 2004 Nematode molecular diagnostics: from bands to barcodes. Annual Review of Phytopathology 42:367–383. 10.1146/annurev.phyto.42.040803.140348 15497206

[ref032] Sambuo D. 2015 Causality analysis of sugar productions and consumption in Tanzania. Journal for Studies in Management and Planning 1:511–522.

[ref033] Sikora R. A., Coyne D., Hallmann J. and Timper P. 2018 Plant parasitic nematodes in subtropical and tropical agriculture CABI, Boston.

[ref034] Singh P. R., Couvreur M., Decraemer W. and Bert W. 2019 Survey of slug-parasitic nematodes in East and West Flanders, Belgium and description of *Angiostoma gandavensis* n. sp. (Nematoda: Angiostomidae) from arionid slugs. Journal of helminthology 94, 11pp. 10.1017/S0022149X19000105 30761968

[ref035] Singh P. R., Nyiragatare A., Janssen T., Couvreur M., Decraemer W. and Bert W. 2018 Morphological and molecular characterisation of *Pratylenchus rwandae* n. sp. (Tylenchida: Pratylenchidae) associated with maize in Rwanda. Nematology 20:781–794.

[ref036] Songela F. and Maclean A. (2008), “Scoping exercise (situation analysis) on the biofuels industry within and outside Tanzania”, Energy for Sustainable Development report for the WWF Tanzania Programme Office, Kinondoni.

[ref037] Steven A., Sunday S. and Fisayo D. 2014 Biodiversity of plant-parasitic nematodes of sugarcane in Bacita, Nigeria. Journal of Entomology and Nematology 6:71–79.

[ref038] Stirling G. R. and Blair B. L. 2001 Nematodes are involved in the yield decline syndrome of sugarcane in Australia. Proceedings of the Australian Society of Sugar Cane Technology 24:430–433.

[ref039] Subbotin S. A., Akanwari J., Nguyen C. N., del Prado Vera I. C., Chitambar J. J., Inserra R. N. and Chizhov V. N. 2017 Molecular characterisation and phylogenetic relationships of cystoid nematodes of the family Heteroderidae (Nematoda: Tylenchida). Nematology 19:1065–1081.

[ref040] Sulle E., Smalley R. and Malale. L. 2014 Opportunities and challenges in Tanzania’s sugar industry: lessons for SAGCOT and the new alliance. Policy brief 76 Future Agricultures Consortium and PLAAS, Brighton.

[ref041] Sundararaj P. and Mehta U. K. 1993 Patterns of interspecific associations in plant parasitic nematodes of sugarcane ecosystems. Nematologia Mediterranea 21:275–277.

[ref042] Tarimo A. J. P. and Takamura Y. T. 1998 Sugarcane production, processing and marketing in Tanzania. African Study Monographs 19:1–11.

[ref043] Tobar Jiménez A. 1969 Descripcion del *Tylenchorhynchus ventrosignatu*s n. sp. (Nematoda: Tylenchida). Revista Iberica Parasitologia 29:349–403.

[ref044] Van den Berg E. 1978 The genus *Rotylenchulus* Linford and Oliveira, 1940 (Rotylenchulinae: Nematoda) in South Africa. Phytophylactica 10:57–64.

[ref045] Van den Berg E., Palomares-Rius J. E., Vovlas N., Tiedt L. R., Castillo P. and Subbotin S. A. 2016 Morphological and molecular characterisation of one new and several known species of the reniform nematode, *Rotylenchulus* Linford & Oliveira, 1940 (Hoplolaimidae: Rotylenchulinae), and a phylogeny of the genus. Nematology 18:67–107.

[ref046] Vrain T. C., Wakarchuk D. A., Levesque A. C. and Hamilton R. I. 1992 Intraspecific rDNA restriction fragment length polymorphism in the *Xiphinema americanum* group. Fundamental and Applied Nematology 15:563–573.

[ref047] Whitehead A. G. and Hemming J. R. 1965 A comparison of some quantitative methods of extracting small vermiform nematodes from soil. Annals of Applied Biology 55:25–38.

[ref048] Williams J. R. 1960a Studies on the nematode soil fauna of sugar cane fields in Mauritius. 4. Tylenchoidea (partim). Occas. Paper. Mauritius Sugar Industry Res. Inst 4:1–30.

[ref049] Williams J. R. 1960b. Studies on the nematode soil fauna of sugarcane fields in Mauritius 5. Notes upon a parasite of root-knot nematodes 1. Nematologica 5:37–42.

